# Propofol-Induced miR-493-3p Inhibits Growth and Invasion of Gastric Cancer through Suppression of DKK1-Mediated Wnt/*β*-Catenin Signaling Activation

**DOI:** 10.1155/2023/7698706

**Published:** 2023-01-31

**Authors:** Kaishuai Zhan, Xiaodan Song, Qian Zhang, Jianshu Yang, Shoutang Lu

**Affiliations:** ^1^Department of Anesthesiology, The Third Affiliated Hospital of Shandong First Medical University, Jinan, 250031 Shandong, China; ^2^Department of Gastrointestinal Surgery, The Third Affiliated Hospital of Shandong First Medical University, Jinan, 250031 Shandong, China

## Abstract

**Purpose:**

Gastric cancer (GC) is one of the most common malignant tumors and also one of the most deadly tumors. In recent years, studies have shown that propofol can inhibit the proliferation and metastasis of many tumor cells. In the present study, we aimed to investigate the underlying mechanism of propofol inhibition of the growth and invasion of GC cells.

**Methods:**

Human gastric cancer cell line SGC-7901 and human normal gastric epithelial cell GES-1 were cultured in high-glucose Dulbecco's Modified Eagle's Medium (DMEM) supplemented with 10% fetal bovine serum at 37°C with 5% CO_2_. Propofol of different concentrations (0, 2, 5, and 10 *μ*g/mL) was used to treat SGC-7901, and miR-493-3p inhibitor was transfected into SGC-7901. The cell proliferation of SGC-7901 was analyzed by MTT as well as colony formation assay. The qRT-PCR was used to assess the expression of mRNA for key genes. We examined the protein expression of DKK1 and relative markers with western blot. Putative binding places of miR-493-3p on the 3′-untranslated area of DKK1 were predicted using bioinformatics and dual-luciferase method.

**Results:**

Propofol prohibited phenotypic features of GC, according to our findings. Furthermore, research into the underlying mechanisms of propofol's suppressive effects in GC cell proved that propofol therapy improved the degrees of expression of the potential tumor suppressor miR-493-3p. The inhibiting properties of propofol on GC cell development, migration, and invasion were abolished when propofol-induced miR493-3p was silenced with anti-miR-493-3p. We also found that this drug reversed epithelial-mesenchymal transformation in SGC-7901 cells via inducing miR-493-3p. Propofol-induced miR-493-3p decreases GC cell development via targeting DKK1 and hence inhibits Wnt/*β*-catenin signaling, according to these findings.

**Conclusion:**

Propofol-induced miR-493-3p decreased GC cell development via targeting DKK1 and hence inhibited Wnt/*β*-catenin signaling, according to these findings.

## 1. Introduction

One of the most frequent gastrointestinal cancers is gastric cancer (GC), with the fifth-highest incidence of cancer in the world and the fourth-highest number of cancer-related deaths [1]. However, despite resection, the prognosis for patients with gastric cancer remains poor, owing to the high likelihood of recurrence and metastasis. Its rapid induction of anesthesia and fast recovery from anesthesia make propofol (2,6-diisopropylphenol) a popular intravenous anesthetic. In clinical trials, propofol-based total intravenous anesthesia for surgical resection of various tumors has been showed to decrease the possibility of recurrence. Zhang et al. found that paravertebral block-regional anesthesia with propofol might be beneficial for reducing locoregional recurrence in women with breast cancer receiving BCS compared with inhalational general anesthesia without propofol [2].

It has been established that propofol inhibits various kinds of cancers, including pancreatic cancer, ovarian cancer, and gastric cancer [3–6], by triggering apoptosis and by inhibiting proliferation, motility, and invasion of cancer cells. Through modulation of RhoA-dependent integrin clustering and actin stress fiber formation, propofol can effectively inhibit the invasion of human cancer cells in vitro at clinically relevant concentrations. Propofol can prevent the invasion of human cancer cells in vitro at therapeutically relevant dosages via modulating RhoA-dependent clumping of integrins and synthesis of actin stress fibers [7]. Human pancreatic cancer cells were induced to undergo apoptosis after being treated with propofol, which inhibited miR-21/Slug signaling. Also, propofol stimulation activated anti-infective and anticancer immunity as well as the differentiation of T-helper cells. However, the mechanism of propofol inhibiting the proliferation, migration, and invasion of cancer cells remains unclear.

MicroRNA (miR) is a short-stranded noncoding RNA of about 22-25 bp in length, which can target the mRNA that binds to the target gene through specific conserved sequences and promote its degradation to inhibit the posttranscriptional target gene's expression, thus participating in the regulation of cellular signaling pathways and playing an oncogenic or procancer role [8]. The overexpression of miR-27a, for example, has been observed to boost GC cell proliferation and invasion, and miR-3148 inhibits the migration of GC NCI-N87 cells by affecting the RRM2 expression. It could therefore serve as a useful way for treating cancers to find agents that upregulate tumor suppressors or miRNAs or downregulate tumor-specific miRNAs. miR-493-3p has been shown to serve as a tumor suppressor in ovarian cancer [9] , lung cancer [10], and breast cancer factor [11]. However, the role of miR-493-3p in gastric cancer was not clear yet.

Our previous results showed that the miR-493-3p expression increased in gastric cancer cells treated with propofol. Propofol was hypothesized to regulate the expression of miR-493-3p, inhibiting gastric cancer proliferation and invasion.

## 2. Matures and Methods

### 2.1. The Use of Cell Lines and Drugs

Human gastric cancer cell line SGC-7901 (Item No. BNCC100114) was purchased from BNCC Cell Bank, and human normal gastric epithelial cell GES-1 (Item No. CBP60512) was purchased from Nanjing Kebai Biotechnology Co. A high-glucose Dulbecco's Modified Eagle's Medium (DMEM) supplemented with 10% fetal bovine serum (FBS; Life Technologies) at 37°C with 5% CO_2_ was routinely used. Sigma-Aldrich provided propofol, which was dissolved in DMEM. To achieve final concentrations of 0, 2, 5, and 10 *μ*g/mL for trials, propofol was diluted with a full culture material.

### 2.2. Transient Transfection

Guangzhou RiboBio developed and manufactured the miR-493-3p inhibitor and its negative control (RiboBio Inc., Guangzhou, China). At 30 to 50 percent confluence, the cells were planted into six-well plates. Then, using Lipofectamine 2000 reagent, cell transfection was carried out according to instructions. After 48 to 72 h, these cells were collected for next trials.

### 2.3. MTT Assay

The MTT assay was used to measure cell viability. The cells were plated at a density of 1 × 10^3^ cells/well onto 96-well plates. The cells were maintained with 0, 2, 5, and 10 g/mL of propofol for 1, 2, 3, and 4 days, respectively, after overnight incubation at 37°C. Cells were transfected with miR-493p inhibitor prior to propofol and cultured for 1, 2, 3, and 4 days for the propofol plus miR-493p inhibitor combo treatment. 20 *μ*L of MTT solution was applied to every matching well at each given time point and was incubated for 4 h. The formed formazan crystals were dissolved with 150 *μ*L dimethyl sulphoxide and then quantified using a plate reader at 490 nm excitation.

### 2.4. Plate Clone Formation Assay

Cells were seeded in 48-well plates. After transfection for 48 h, the old culture medium was discarded and washed with phosphate buffer (PBS). The digestion solution and culture medium were successively added to prepare the cell suspension (3 × 10^4^/mL). The cell suspension was seeded in 12-well plates, and the culture medium was replaced every 3 d. The number of clonal cells was observed under microscope.

### 2.5. Cell Migration and Invasion Assay

Cancer cell movement was measured using transwell migration assays. Gastric cancer cells were seeded onto transwell chambers with four amounts of propofol treatment or anti-miR-493-3p transfected gastric cancer cells after propofol (5 g/mL) therapy (Corning Incorporated, Corning, NY). After 24 h of treatment, these GC cells were collected and washed twice with PBS to remove the serum. The cells were resuspended at a density of1 × 10^5^cells/well in 100 mL serum-free media and put at the top of the device. The bottom chamber was also supplemented with media containing 10% FBS. After 15 h, the migratory cells attached to the lower side of the membranes insertion were preserved, dyed with Giemsa, and photographed under a microscope.

### 2.6. Luciferase Assay

The StarBase database, RNAInter, Jefferson, and miRDB websites were utilized to determine the locations of the binding sites for miR-493-3p and DKK1. To make the wild-type (DKK1-wt) and mutant-type (DKK1-mut) luciferase plasmids, the binding and mutation areas were cloned into the luciferase vector pGL3. SGC-7901 cells (2 × 10^5^ cells/well) were plated in 6-well dishes and incubated for 24 h. The NC or miR-493-3p luciferase vectors were cotransfected into SGC-7901 cells using Lipofectamine 2000 according to the manufacturer's instructions. After 24 h of transfection, luciferase activity was measured using the dual-luciferase reporter assay kit (Beijing Solar Science & Technology Co., Ltd.). The cell tests were carried out three times in a row.

### 2.7. Western Blot Analysis

Gastric cancer cells (5 × 10^5^/well) treated either with or without anti-miR-493-3p were planted into six-well plates and given a 48-hour propofol (5 *μ*g/mL) therapy. All materials were recovered from these cells using RIPA lysis buffer containing protease inhibitors (Sigma-Aldrich). Total proteins were then retrieved from these cells using RIPA lysis buffer with enzyme inhibitors (Sigma-Aldrich). By electrophoresis on a 10% SDS polyacrylamide gel, equal quantities of all protein (20 *μ*g) were extracted and put into a PVDF membrane. After that, membrane was masked for 2 h with 5% nonfat milk before being treated with the specified antibodies. The protein formation was observed using increased fluorescent dye reagents (Merck Millipore, Darmstadt, Germany) after subsequent incubation with horseradish peroxidase (HRP). A ChemiDoc CRS + Molecular Imager (BioRad, Hercules, CA) was used to take the images.

### 2.8. Real-Time Quantitative PCR

The genes of interest were quantified using real-time PCR (RT-PCR). Specifically, gastric cancer cells were treated with 0, 2, 5, and 10 *μ*g/mL propofol, or 5 *μ*g/mL propofol was used to treat cells transfected with anti-miR-493-3p. All RNA was isolated from cells using TRIzol and then subjected to RT-PCR with specified primers and SYBR Green as a fluorescent dye to analyze the genes of interest. RT-PCR was carried out utilizing a BioRad real-time PCR machine, and the primers were listed in Table 1. As internal controls, U6 and *β*-actin genes were employed, respectively. The relative gene expression was measured by the 2^-*ΔΔ*Ct^ method.

### 2.9. Statistical Analysis

All data were analyzed using the SPSS 17.0 statistical software, and results were presented as mean ± SD. The Student *t*-test for two groups, one-way analysis of variance for multiple groups, and parametric generalized linear model with random effects for the MTT assay were all judged statistically significant at *P* < 0.05. The data sets were all two-sided, with asterisks indicating statistically significant results (^∗^*P* < 0.05 and ^∗∗^*P* < 0.01, respectively).

## 3. Results

### 3.1. In Vitro Propofol Inhibited SGC-7901 Cell Growth and Migration while Increasing the Expression of miR-493-3p

**Figure 1:**
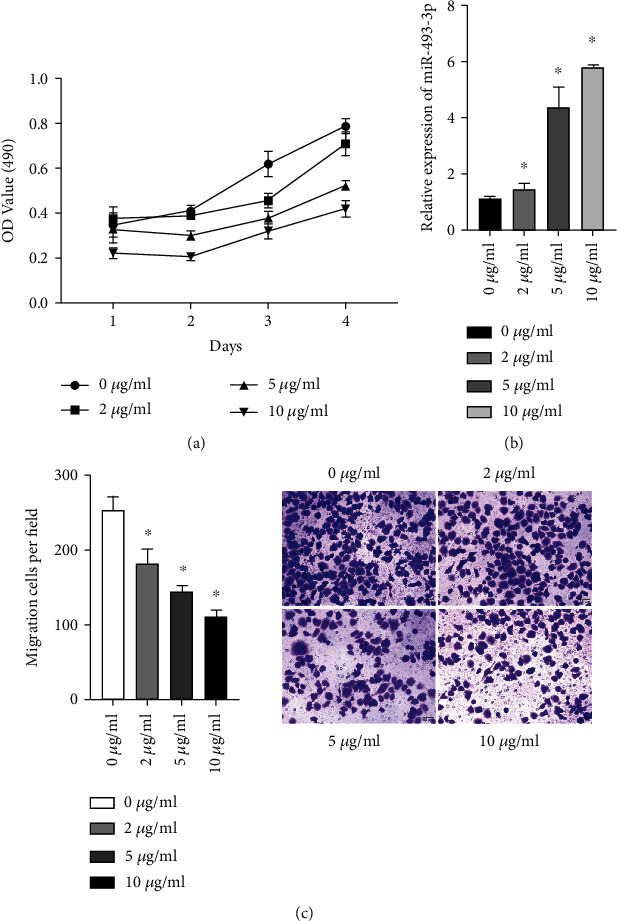
The inhibitory effects of propofol on the proliferation and migration of GC cells in vitro. (a). The cell proliferation was measured by the MTT assay. (b). Propofol treatment increased the expression levels of miR-493-3p on gastric cancer cells, as measured by qRT-PCR. (c). After treatment with propofol (0, 2, 5, and 10 *μ*g/mL) for 48 h, the cell migration ability of gastric cancer cells was inspected for transwell assays. GC: gastric cancer; qRT-PCR: real-time quantitative polymerase chain reaction. ^∗^*P* < 0.05.

To detect the effect of isoproterenol on GC cell, we initially looked at how isoproterenol affected stomach cell growth and invasion. MTT results showed that isoproterenol treatment significantly reduced the proliferation of SGC-7901 cells in a dose- and time-dependent manner (Figure 1(a)). As a result, our findings imply that drug inhibits the proliferation and invasion characteristics of SGC-7901 cells.

The underlying mechanism of propofol's inhibitory impact on GC cells was then studied. Intriguingly, we discovered that isoproterenol administration raised the levels of the expression of miR-493-3p in SGC-7901 tumor cell line manner. When compared to untreated cells, the levels of miR-493-3p in GC cell lines increased more than 2-fold at 5 *μ*g/mL propofol and more than 3-fold at 10 *μ*g/mL propofol (Figure 1(b)). In addition, propofol obviously inhibited cell migration in gastric cancer (Figure 1(c)).

### 3.2. Propofol's Inhibitory Effects on Gastric Cancer Cells Were Substantially Abolished by Anti-miR-493-3p Transfection

**Figure 2:**
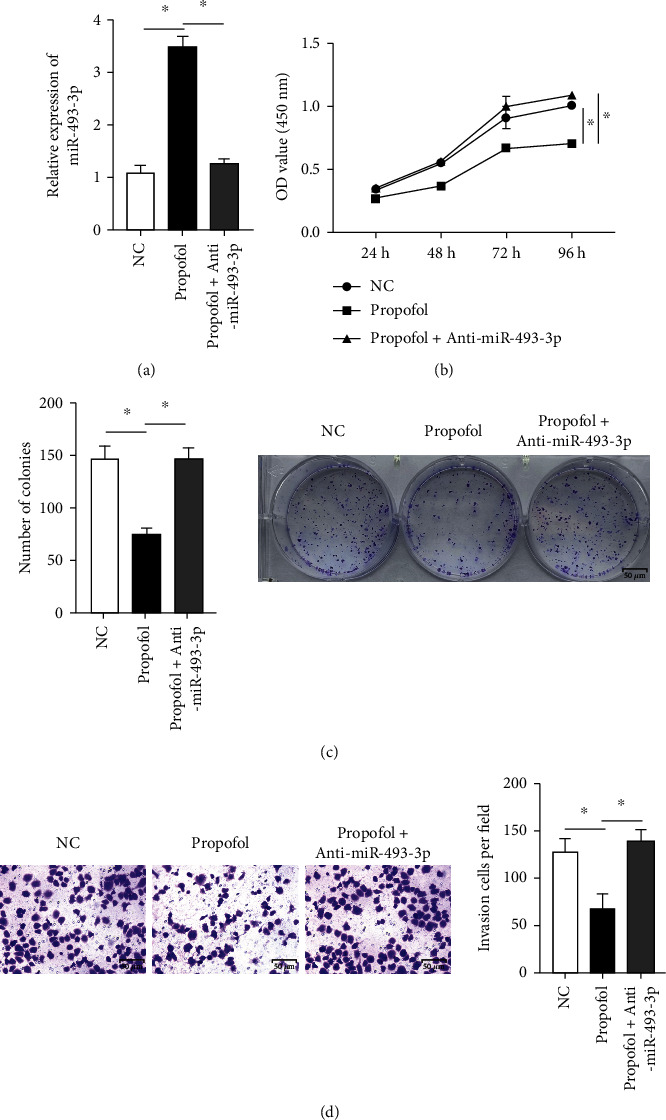
Anti-miR-493-3p transfection reversed the inhibitory effects of propofol on cell proliferation and migration of GC cells. (a) The expression of miR-493-3p in SGC-7901 cell was measured by qRT-PCR. (b, c) Anti-miR-493-3p transfection abrogated the inhibition of propofol on the proliferation of GC cells as measured by the MTT (b) and colony-forming (c) assays. (d) Anti-miR-493-3p transfection suppressed the inhibitory effects of propofol on the invasion capacity of GC cells, which was measured by transwell assays. GC: gastric cancer; qRT-PCR: real-time quantitative polymerase chain reaction. ^∗^*P* < 0.05.

Propofol seems to inhibit gastric cancer cells by upregulating miR-493-3p, according to the aforementioned findings. The next step was to see if the suppressing propofol-induced miR-493-3p expression using anti-miR-493-3p transfection eliminated propofol's inhibitory effects in gastric cancer cells. Anti-miR-493p transfection successfully lowered miR-493p expression levels, according to real-time quantitative PCR (RT-PCR) research (Figure 2(a)). In contrast to prior findings, anti-miR4933p transfection dramatically inhibited propofol-induced suppression of gastric cancer cell growth in colony-forming and MTT assay (Figures 2(b) and 2(c)). Furthermore, transwell showed that anti-miR-493-3p transfection effectively inhibited propofol's inhibitory effects on gastric cancer cell invasion and metastasis (Figure 2(d)).

### 3.3. Validation of the Targeting Relationship between miR-493-3p and DKK1

**Figure 3:**
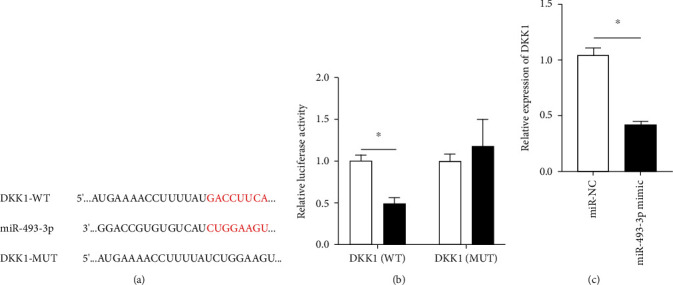
miR-493-3p targeted DKK1. (a) Binding sites of miR-493-3p and DKK1 were predicted through the StarBase website. (b) Binding association of miR-493-3p and DKK1 was verified by the dual-luciferase reporter assay. (c) The DKK1 expression was detected by qRT-PCR. The cell experiment was repeated three times independently. ^∗^*P* < 0.05. GC: gastric cancer; qPCR: quantitative polymerase chain reaction.

TargetScan software predicts a complementary binding place between miR-493-3p and DKK13′UTR (Figure 3(a)). The dual-luciferase reporter gene assay showed that the luciferase activity of SGC-7901 cells in the miR-493-3p + Wt-DKK1 group was significantly lower than that of the miR-control+Wt-DKK1 group, but the difference in luciferase activity between miR-control+Mut-DKK1 and miR-493-3p + Mut-DKK1 groups was not statistically significant (Figure 3(b)). miR-493-3p mimic obviously decreased the DKK1 expression (Figure 3(c)).

### 3.4. Propofol-Induced miR-493-3p Reduced SGC-7901's Epithelial-Mesenchymal Transition via Suppressing DKK1/Wnt/Catenin Signaling

**Figure 4:**
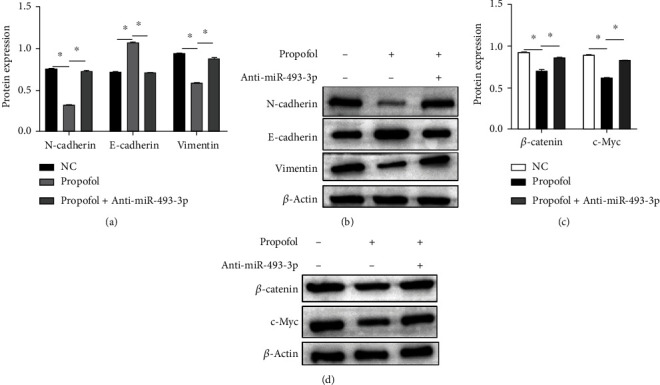
Propofol-induced miR-493-3p targeted DKK1 and attenuated Wnt/*β*-catenin signaling in GC cells. (a, b) The expression of EMT markers (E-cadherin, N-cadherin, and vimentin) was measured by western blot analysis. (c, d) Western blot analysis was used to assay the expression of DKK1, *β*-catenin, and c-Myc. EMT: epithelial-mesenchymal transition; GC: gastric cancer. ^∗^*P* < 0.05.

As demonstrated in Figures 4(a) and 4(b), isoproterenol administration raised the expression of miR-493-3p, as well as enhanced the level of the epithelial indicator E-cadherin and reduced the level of the mesenchymal indicators N-cadherin and vimentin at the mRNA and protein levels. SGC-7901 cells treated with anti-miR-493-3p, on the other hand, reversed propofol's inhibition of EMT.

DKK1 has been discovered as a direct miR-493-3p downstream protein. DKK1 has also been demonstrated to increase the activity of Wnt/-catenin pathway in gastric cancer cells in prior investigation [12], suggesting that propofol inhibited Wnt/*β*-catenin pathway. Isoproterenol treatment significantly reduced expression levels of *β*-catenin and c-Myc (Figures 4(c) and 4(d)). Finally, these findings imply that propofol-induced miR-493-3p suppressed EMT in gastric cancer cells by inhibiting DKK1-mediated activation of Wnt/*β*-catenin pathway.

## 4. Discussion

Propofol is a sedative-hypnotic drug given intravenously to continue to keep anesthesia. In addition, it has been shown to protect neurons from ischemia in the brain [13]. Many trials have shown that this drug has various characteristics, including anticancer [14], antiproliferative [15], anti-inflammatory [16], and antioxidant [17]. Isoproterenol has been shown to reduce the toxic potential of pancreatic and lung cancer cells [18, 19]. Similarly, isoproterenol has been reported to induce apoptosis in gastric cancer cells through activation of Bax and caspase-3. We found that isoproterenol treatment inhibited GC cell by regulating DKK1-mediated activation of Wnt/*β*-catenin pathway, resulting in upregulation of miR-493-3p. In recent decades, miRNAs have been demonstrated to play a key role in the development of a variety of malignancies, including GC cells [20]. Although isoproterenol has been reported to have an inhibitory effect on gastric cancer cells, no increase in level of miR-493-3p was discovered as a result of isoproterenol treatment. In cancer patients, the reduced expression of miR-493-3p is linked to bigger tumors, worse division, and shorter survival periods [21–23]. These studies suggested that miR-493-3p might serve as a candidate tumor suppressor in gastric cancer. Indeed, the overexpression of miR-493-3p could prevent cancer cells from proliferating and invading. As a result, we believe that the enhanced expression of the putative tumor suppressor miR-493-3p is responsible for propofol's inhibitory action on gastric cancer cells.

miRNAs attach to the 3′ untranslated region of target mRNAs and suppress the translation of the associated gene. By targeting lymphoid enhancer-binding factor 1, miR-493-3p reduced colorectal cancer EMT and metastasis.

DKK1 has been identified as a target of miR-493-3p in many studies. According to these findings, propofol-induced enhanced production of miR-493-3p should reduce the DKK1 expression in gastric cancer cells. The isoproterenol-mediated expression of miR-493-3p downregulated the expression level of DKK1, and miR-493-3p was found to stop the cell cycle of gastric cancer cells at the G1 phase by inhibiting DKK1. DKK1 is a cell surface protein that is significantly expressed in a number of human malignancies, including breast [24], lung [25], rectal, and gastric cancers [26, 27]. DKK1 is also associated with survival outcomes in gastric cancer, with high DKK1 expression or high DKK1 expression with *β*-catenin positivity being an independent factor in poorer overall (OS) (*P* < 0.05) and disease-free survival (DFS) in gastric cancer patients [28]. DKK1 has also been connected to the stimulation of several signaling pathways in gastric cancer cells that enhance tumor formation [29, 30]. Among these pathways, Wnt/-catenin signaling has received increased attention as it plays a key role in gastric carcinogenesis and progression. DKK1 activates the Wnt pathway, which promotes the development of gastric cancer cells. As a result, our findings reveal that inhibiting DKK1 via isoproterenol-mediated miR-493-3p production could suppress Wnt/-catenin signaling activation.

In the evolution of gastric cancer cells, aberrant Wnt signaling has been identified most frequently. The stimulation of the Wnt/catenin-mediated signaling cascade may have a critical role in gastric cancer, according to the findings [31–33]. The development of gastric cancer cells was successfully suppressed by pharmacological suppression of Wnt signaling [34]. Furthermore, cancer cells with an EMT-like phenotype had dysregulated stimulation of the Wnt/catenin pathway, which gave the cancer cells highly metastatic capabilities [35, 36]. In Vierge County cells, inhibition of the Wnt pathway by II-spectrin suppressed EMT and invasion. Based on earlier research, we believe that propofol's ability to reverse EMT in gastric cancer is due to its suppression of the Wnt/-catenin pathway. Given together, we believe that isoproterenol-mediated regulation of DKK1 by miR-493-3p may impair Wnt/-catenin pathway, resulting in the reduction of gastric cancer development, migrating, and EMT.

Overall, our trials showed that propofol inhibited stomach cancer cell growth, invasion, and migratory. The influence may be obtained by upregulating the expression of miR-493-3p, which suppressed DKK1-mediated stimulation of Wnt/*β*-catenin pathway and subsequently regulated gastric cancer development, and migration and EMT.

## Figures and Tables

**Table 1:** The primers used in this study.

Primers' name		Sequence (5′-3′)
DKK1	Forward	TCGGCGGCAGTAAGAAGG
	Reverse	TGTGGGCTAAGTCAAATGAAGTG
miR-493-3p	Forward	UGAAGGUCUACUGUGUGCCAGG
	Reverse	CCUGGCACACAGUAGACCUUCA
U6 snRNA	Forward	TGCGGGTGCTCGCTTCGGCAGC
	Reverse	GTGCAGGGTCCGAGGT

## Data Availability

Data to support the findings of this study is available on reasonable request from the corresponding author.
